# Evidence underscoring immunological and clinical pathological changes associated with *Sarcoptes scabiei* infection: synthesis and meta-analysis

**DOI:** 10.1186/s12879-022-07635-5

**Published:** 2022-07-28

**Authors:** Christina Næsborg-Nielsen, Vicky Wilkinson, Natalia Mejia-Pacheco, Scott Carver

**Affiliations:** Department of Biological Sciences, University of Tasmania, Private Bag 55, Hobart, TAS Australia

**Keywords:** *Sarcoptes scabiei*, Immunology, Pathology, Sarcoptic mange, Scabies, Meta-analysis

## Abstract

**Background:**

*Sarcoptes scabiei* is one of the most impactful mammalian parasites. There has been much research on immunological and clinical pathological changes associated with *S. scabiei* parasitism across a range of host species. This rich body of literature is complex, and we seek to bring that complexity together in this study. We first (1) synthesise narrative reviews of immunopathological relationships to *S. scabiei* infection to construct overarching hypotheses; then (2) undertake a systematic meta-analysis of primary literature on immunological and clinical pathological changes; and lastly (3) contrast our findings from the meta-analysis to our synthesis from narrative reviews.

**Methods:**

We synthesised 55 narrative reviews into two overarching hypotheses representing type I and type IV immune responses to *S. scabiei* infection. We then systematically extracted all literature reporting immunological variables, acute phase proteins, oxidant/antioxidant status, and erythrocytic, hepatological and nephrological changes, calculating 565 effect sizes between controls and sarcoptic mange affected groupings, refining (simplifying) hypotheses from narrative reviews.

**Results:**

Immunological and clinical pathological parameters were most often studied in dogs (n = 12) and humans (n = 14). Combining immunological and clinical pathological information across mammalian species (n = 19) helped yield general insights into observed disease responses. This is evidenced by interspecific consensus in 27 immunological and clinical pathology variables (6/26 type I hypersensitivity, 3/20 type IV hypersensitivity, 6/10 oxidant/antioxidant status, 3/6 acute phase protein, 4/7 erythrocytic, and 5/10 hepatological/nephrological).

**Conclusions:**

Elevated IgE, eosinophils and mast cells in type I hypersensitivity response corresponded to what was described in narrative reviews. Results from type IV hypersensitivity response suggested typical antibody response, however cell-mediated response was less evident. Some consensus of acute phase protein response and shifted oxidant/antioxidant balance and slight evidence of anemia. We highlight the need for mange/scabies studies to more routinely compare immunological and clinical pathological changes against controls, and include collection of a more standardised suite of variables among studies.

**Supplementary Information:**

The online version contains supplementary material available at 10.1186/s12879-022-07635-5.

## Introduction

*Sarcoptes scabiei* is one of the most impactful of mammalian parasites [1]. It is documented to infest nearly 150 species, and is a Neglected Tropical Disease of humans with prevalence of approximately 100 million cases [2–4]. Infection with this parasitic mite causes the disease ‘scabies’ in humans, which is termed ‘sarcoptic mange’ in non-human animals (hereafter, ‘mange’) [5]. Mange has been recognised since antiquity [6], and knowledge of the mechanisms that dictate disease severity has grown substantially over the last two decades, creating a complex body of literature on the associated immunological and clinical pathology variables.

Clinical pathological signs of mange are driven by host immune responses to the mite. Physical signs range from mild erythema to the development of severe dermatitis, hyperkeratotic crusts, alopecia and progressive systemic disease characterised by weight loss, secondary bacterial infections [7–9] and death [10]. A fascinating aspect of mange is that pathological outcomes broadly fall into two categories, depending on the type of hypersensitivity reaction mounted by the host [5]: a Type I immediate antibody-mediated immune response [4, 11–13]; or Type IV delayed cell-mediated immune response [14–17]. Type I reactions are typified by the Immunoglobulin(Ig) E-mediated activation of mast cells and eosinophils, whereas Type IV is associated with sensitised T cells that either cause damage directly or activate other leukocytes [18] (see Additional file 1: S1 and Additional file 3: S3 Appendicies). Such responses are often referred to as immunopathological because of the innapropriate immune response to the infection that can cause harm to the host. Intra- and interspecific variation is observed in the type and magnitude of the hypersensitivity reaction that develops. In some hosts, such as humans and domestic dogs, Type I hypersensitivity is the most frequently observed, with disease termed ‘ordinary’ mange with pathological and clinical signs of mild to intense pruritus and alopecia. However, other hosts, such as wombats, canids (e.g., red foxes, raccoon dogs and San Joaquin kit foxes), and immunocompromised humans, appear predisposed to developing a Type IV hypersensitivity reaction, resulting in a more severe form of disease known as ‘crusted’ mange/scabies [15] with signs of severe hyperkeratosis and crusts.

The immunological and clinical pathological changes associated with hypersensitivity reactions to *S. scabiei* infection have been subject to much research across host species [19–46]. Several narrative reviews have brought together the complex array of empirical studies into excellent syntheses, albeit mostly focused on pathology observed in humans [7, 13, 17, 47, 48] or a single species [4, 49]. However, a synthesis of mange associated changes in immunology and clinical pathology, that is both empirical and brings together data from across a compilation of host species, is yet to be undertaken. Meta-analysis provides a useful way to synthesise *S. scabiei* studies, comparing the effect of infected groups with uninfected individuals, as well as across observed disease severity from the same experiment. By bringing together empirical studies on *S. scabiei* from across host species in a formal analysis framework, a deeper understanding of the interspecies (dis)similarities in the pathology of mange may be developed to the benefit of all hosts, as well as highlight potential knowledge gaps in disease pathology.

In this research, we undertake a comprehensive empirical examination of reported immune, erythrocytic and biochemical parameters observed in host species with clinical signs of mange to give as holistic a view as possible. We synthesise both narrative reviews and empirical studies to describe the immunological and clinical pathological changes occurring in hosts affected by *S. scabiei*. We include information on all reported variables in the literature, comprising of immunological variables, acute phase proteins (APPs), oxidant/antioxidant status, and erythrocytic, hepatological and nephrological changes. We have three overarching objectives: (1) synthesise existing narrative reviews of immunopathological relationships to *S. scabiei* infection and construct overarching hypotheses of immunopathological relationships based on these reviews; (2) undertake a systematic meta-analysis of primary literature on immunological and pathological changes observed in association with scabies/mange; and (3) utilise the formal meta-analysis to describe immunological and clinical pathological changes with comparison to the literature from Objective 1.

## Methods

### Synthesising narrative reviews of sarcoptic mange pathology

In October 2020, we sought to capture all narrative literature reviews available on *S. scabiei* infection in online databases Web of Science, PubMed, and Scopus. We used broad search criteria by including variations on the disease name, such as sarcoptic mange, scabies and *Sarcoptes scabiei*, and solely selected to view reviews. Following literature collection, we screened all reviews based on their abstracts and titles to eliminate any review articles not focused on immunology or pathology associated with sarcoptic mange. With the remaining reviews, we synthesised all immunopathological relationships reported and used this to create a conceptual diagram detailing proposed immunopathological relationships associated with infection. The conceptual diagram was created based on the most frequently described immunopathological parameters and relationships reported, with some less commonly mentioned parameters used to draw ‘likely’ connections in a small number of instances (e.g., B cells and some cytokines). Hypersensitivity type was usually listed in the reviews explaining the response to *S. scabiei* infection. In reviews where this was not the case, we categorised the response according to previous knowledge or observations regarding the host’s response, either from other reviews focusing on the same hosts or by screening the observations from the referenced articles.

### Meta-analysis of immunopathological responses associated with *S. scabiei* infection

Between April and October 2020, we undertook a systematic search of the existing literature in the same online databases as mentioned above, to obtain relevant studies reporting the empirical effects of sarcoptic mange on animals and humans. We used the following keywords in two separate searches, which we found best captured both human and non-human animal studies:

(“Sarcoptes scabiei” OR “sarcoptic mange” OR “scabies”) AND (“patho*” OR “physio*” OR “disease” OR “immun*” OR “biochem*” OR “skin” OR “lesion” OR “metabol*” OR “heat” OR “therm*”) AND (“wild*” OR “domestic*”) NOT (“human*” OR “child*” OR “m?n” OR “wom?n” OR “patient*”) NOT (“potato scab” OR “streptomyces”).

(“Sarcoptes scabiei” OR “sarcoptic mange” OR “scabies”) AND (“patho*” OR “physio*” OR “disease” OR “immun*” OR “biochem*” OR “skin” OR “lesion” OR “metabol*” OR “heat” OR “therm*”) AND (“human” OR “child*” OR “m?n” OR “wom?n” OR “patient*” AND “control”) NOT (“potato scab” OR “streptomyces”).

Any variation of the name of the disease was accommodated (e.g., sarcoptic mange or scabies) as well as the name of the parasite (*Sarcoptes scabiei*), and irrelevant diseases (e.g., potato scab or streptomyces) were excluded. Any studies reporting empirical information linking host immunology or pathology with mange/scabies were retained. This resulted in 1394 research articles that were downloaded to an EndNote Library and duplicates were removed. All studies were screened by evaluating the results section, excluding those studies: (i) not mentioning or comparing to a relevant control group; (ii) not informing of sample sizes; (iii) lacking mean values; or (iv) lacking standard deviation (SD) or standard error (SE) values. 63 research articles were included in the final meta-analysis (see Additional file 4: Fig. S1).

For each study, we noted the author, year, parameter(s) examined, key findings, sample sizes across control and treatment groups, hypersensitivity type, host species and country of origin for the study. The mean, SD or SE and sample size for each parameter were recorded to calculate the effect size and confidence intervals (CI). In cases where a control group wasn’t specified, but a time point of sample collecting was, we chose the values the studies used as a ‘base line’ (e.g., healthy, pre-exposure or day 0) as a control group. When multiple means were reported (e.g., separate means for each control subject or males and females separately), the values were pooled using the Cochrane method [50]; the same method was applied for SE and SD.

Mange affected and healthy control groups were compared by the calculation of effect sizes (g) and variance (Vg) using the following equations [51];

$$g = \frac{{x_{1} - x_{2} }}{{S_{within} }}*J\quad V_{g} = \left( {\frac{{n_{1} + n_{2} }}{{n_{1} n_{2} }} + \frac{{d^{2} }}{{2\left( {n_{1} + n_{2} } \right)}}} \right)*J^{2}$$

where x_1_ and x_2_ are the mean values of the control and treatment groups, *n*_1_ and *n*_2_ are sample sizes, *S*_*within*_ is the within-groups standard deviation, and *J* is the correction factor for small sample sizes, $J=1-\frac{3}{4df-1}$. Upper and lower CI was calculated lastly for each effect size using the following equation, $CI=g\pm 1.96*SEg$, where SEg is the square root of the variance (Vg).

**Fig. 1:**
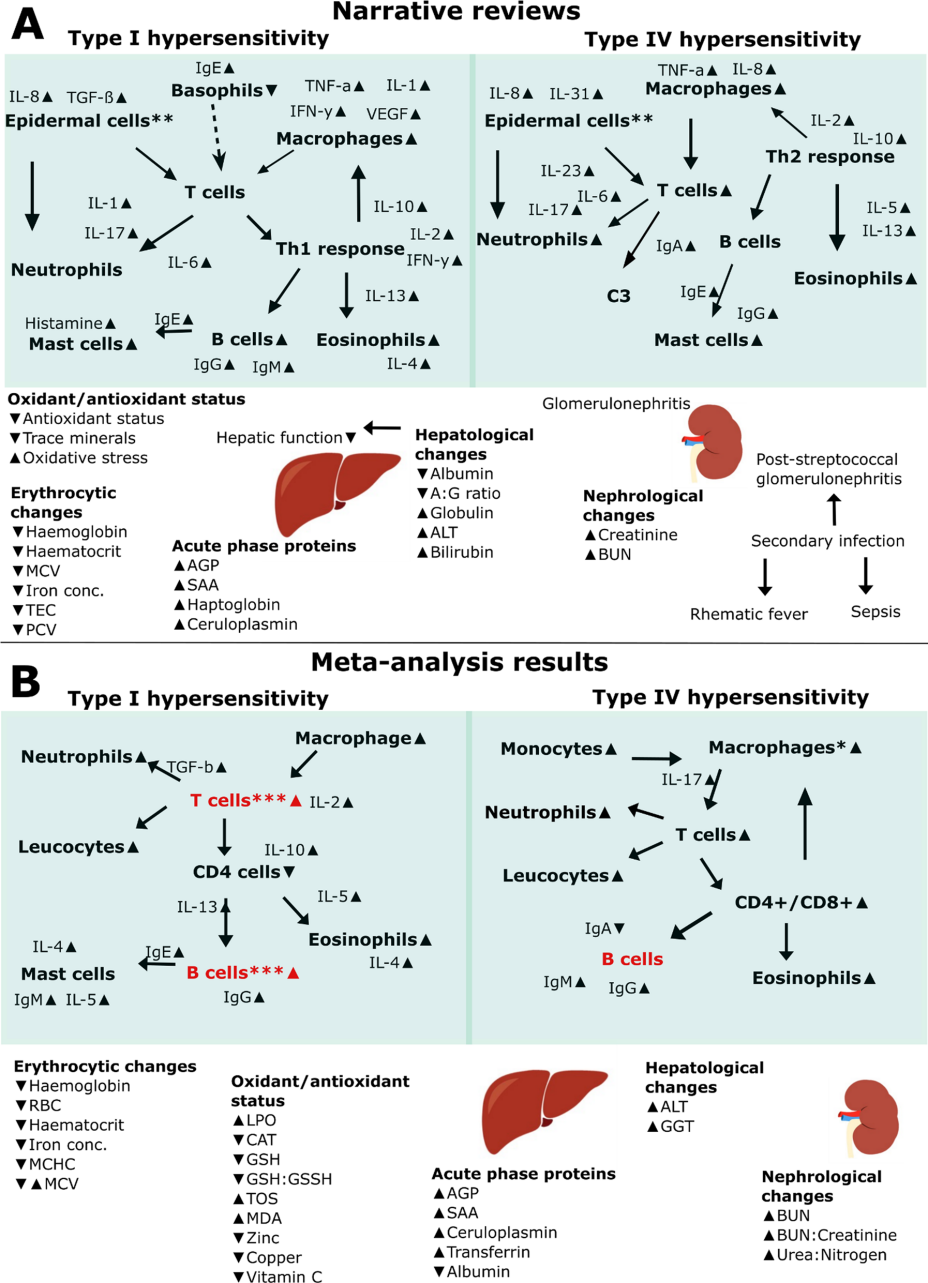
Diagrams of the immunopathological cascades arising from *Sarcoptes scabiei* infection depending on host hypersensitivity response (Type I or IV). Diagram **A** represents the immunopathological processes as currently proposed in narrative literature reviews of *S. scabiei*, and diagram **B** represents the Immunopathological relationships supported by the meta-analysis undertaken in this study. Solid arrow ( →) indicates a stimulation or influence from one parameter to the other, whereas a dashed arrow (– →) indicates a hypothesised link; small up or down triangle next to parameter indicates an increase or decrease; red text indicates missing immunological links considered likely to connect parameters; Parameters in non-bold indicates secreted cytokines or immunoglobulins; *in panel **B** IV indicates no direct measure of macrophages instead measured by MCP-1; **in panel **A** IV indicates epidermal cells to include keratinocytes, Langerhans cells and fibroblasts; *** in panel **B** indicates no direct measure of T cells or B cells however could be included in the measurement of lymphocytes. *IL* interleukin; *IFN-γ* Interferon gamma; *TNF-α* tumour necrosis factor alpha; *TGF-ß* transforming growth factor beta; *CD4+*= T helper cells; *CD8+* cytotoxic T cells; *Ig* immunoglobulin; *C3* complement 3; *MCV* mean corpuscular volume; *TEC* total erythrocyte concentration; *PCV* packed cell volume; *AGP* Acid(1)-alpha glycoprotein; *SAA* serum amyloid A; *A:G ratio* albumin:globulin ratio; *ALT* alanine aminotransferase; *BUN* blood urea nitrogen; *MCHC* mean corpuscular haemoglobin concentration; *MCH* mean corpuscular haemoglobin; *LPO* lipid peroxidation; *CAT* catalase; *GSH:GSSH* free glutathione:oxidized glutathione ratio; *GGT* Gamma-glutamyl transferase. Created in Inkscape

Due to the large number of immunopathological parameters and differences in focus groups with some studies dividing their data based on disease severity (mild/moderate/severe clinical sign presentation) or simply affected/not affected, the data were divided into four different categories against the control group: (a) mange affected; (b) mild cases of mange; (c) moderate cases of mange; and (d) severe cases of mange. By separating our data into these categories, we somewhat reduced the variability observed in study methods. From now on we will refer to category a as ‘mange affected’ or ‘diseased relative to control comparisons; and category b–d as ‘mange severity’. The classification of disease severity was based on the author’s determination or was otherwise categorised based on the percentage of lesions present according to Additional file 4: Table S1. The effect sizes were compared and computed together in R (ver. 1.3.1093) using the metafor package [52]. We investigated whether publication bias existed in the literature for studies reporting immunological parameters for Type I and Type IV hypersentivity, as well as parameters falling into common responses (i.e. oxidant/antioxidant status, acute phase protein response, and erythrocytic, hepatological and nephrological changes). To test for publication bias we plotted standard error against effect sizes for the previous mentioned categories (Additional file 5: Figs. S1–S3). We additionally conducted a regression test to test for data distribution asymmetry using regtest in the metafor package [52]. Finally, a conceptual diagram based on the meta-analysis was created, partially informed by the proposed processes from the narrative reviews in Objective 1, but with only significant relationships (95% Cis not overlapping zero) computed from the meta-analysis added (Fig. 1B).

## Results

### Synthesising narrative reviews of sarcoptic mange-associated clinical pathology

From the initial literature search, 244 narrative review articles were identified. These reviews were diverse, covering topics from socioeconomic impacts of sarcoptic mange to the pathology associated with *S. scabiei*. A conceptual diagram (Fig. 1A) was created based on 55 reviews that primarily described immunological and clinical pathology variables associated with sarcoptic mange (see Additional file 1: S1).

There was an almost equal amount of information regarding Type I and IV hypersensitivity responses (Type I = 32; Type IV = 30). The most common parameters described across studies were eosinophils (Type I, 19 times; Type IV, 20 times), mast cells (Type I, 9 times; Type IV, 5 times) and immunoglobulin E (IgE) (Type I, 16 times; Type IV, 16 times) for both hypersensitivity types. In addition, a Type 1 T helper cell (Th1) response was described 9 times for Type I and 1 time for Type IV, and a Type 2 T helper cell (Th2) response was described 3 times for Type I and 13 times for Type IV hypersensitivity type. Observations from reviews provided evidence of Type I hypersensitivity being associated with IgE activation of basophils and mast cells, and the subsequent release of histamine and other proinflammatory cytokines. Processes documented as part of Type IV hypersensitivity responses included the proliferation of macrophages, neutrophils, and B cells. Antibody responses appeared to be present during both Type I and Type IV hypersensitivity responses, depending on whether there was a Th1 focused response or a Th2 focused response to *S. scabiei* infection. Almost all hosts infested with *S. scabiei* reportedly had altered antioxidant defence, mild to severe anaemia, and hepatological and nephrological abnormalities.

### Publication bias

We chose to look at whether a bias existed in the literature to determine whether the effect sizes we obtained from our analysis were potentially influenced by publication bias. Plotting effect sizes against the standard errors for all immunological parameters for either Type I or Type IV hypersentivity or common responses extracted from the literature resulted in some of the funnel plots with the appearance of largely scattered data points, and plots that appeared asymmetrically distributed (see Additional file 5: Figures). To investigate this further, we conducted an Egger’s regression for funnel plot asymmetry for each category across Type I hypersentivity, Type IV hypersensitivity and common responses. Regressions for Ctrl vs. affected (Type I hypersensitivity: z = 12.60, p < 0.0001; Type IV hypersensitivity: z = 5.79, p < 0.0001; Common responses: z = 4.03, p < 0.0001), mild mange (Type IV hypersensitivity: z = − 4.67, p < 0.0001; Common responses: z = − 6.70, p < 0.0001), moderate mange (Type I hypersensitivity: z = 5.17, p < 0.0001; Type IV hypersensitivity: z = 6.11, p < 0.0001; Common responses: z = − 5.21, p < 0.0001), and severe mange (Type IV hypersensitivity: z = 5.14, p < 0.0001) suggests that a bias does exists (see Additional file 5: Table S1 for full overview).

### Meta-analysis of immunological and clincal pathology changes associated with *S. scabiei* infection

**Fig. 2:**
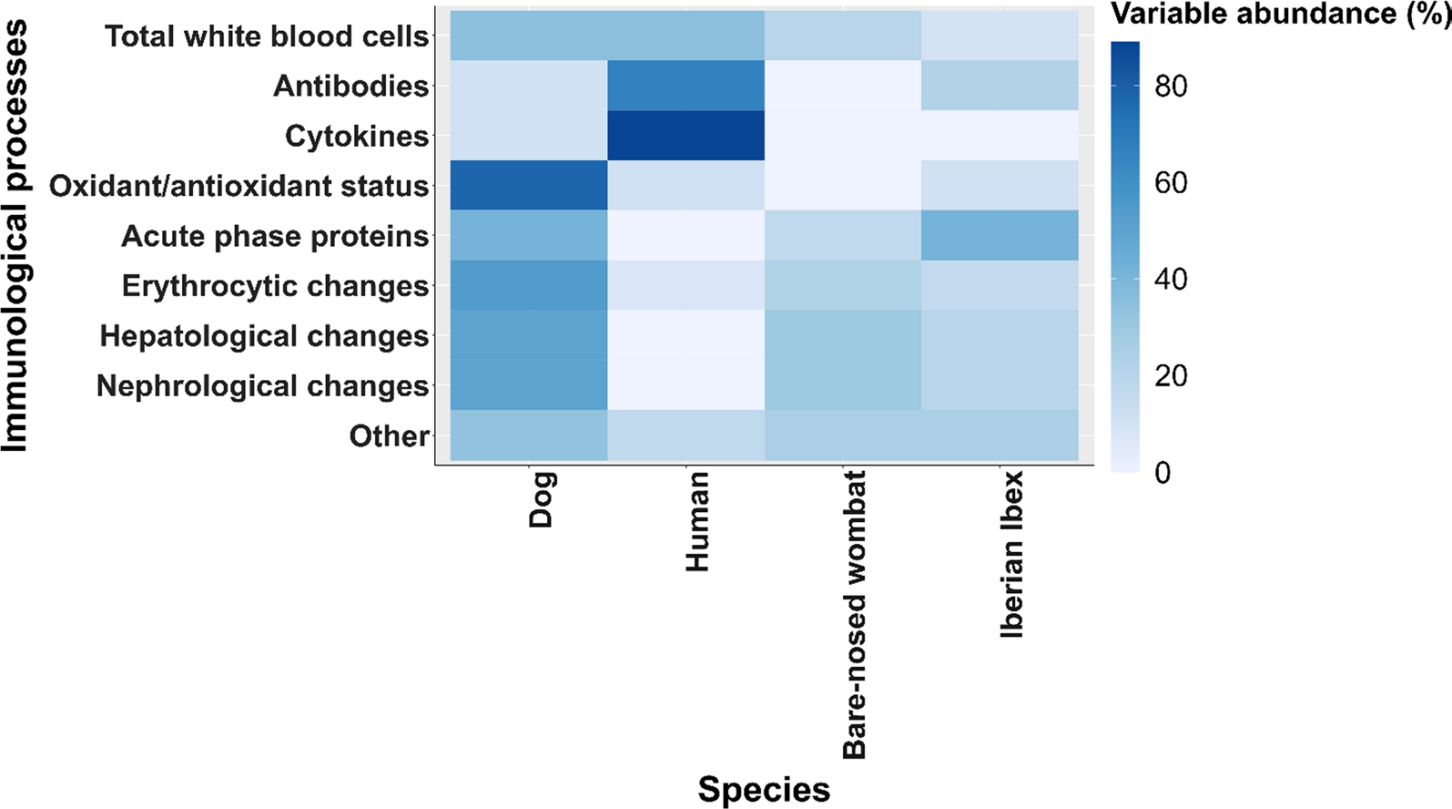
Heatmap illustrating the four host species for which effect sizes were most commonly calculated (dog = 138, human = 76, bare-nosed wombat = 67 and Iberian ibex = 63). The heat reflects the percentage of studies for each category (immunological process) with each category amounting to a total of 100%. Parameters not falling directly into a definitive category, such as ‘Erythrocytic changes or ‘Acute phase proteins’, were included in the category ‘Other’

The full dataset included 565 effect sizes from 63 research articles that reported immune and pathological changes in association with *S. scabiei* infection. 244 effect sizes were categorised as being linked to type I hypersensitivity and 321 were categorised as being linked to type IV hypersensitivity. Neutrophil counts were the most frequently reported parameters (25 times), globulin was the second most reported (23 times) and eosinophils, haematocrit and haemoglobin concentration were the third most reported parameters (21 times each). Data were available for a total of five different orders (Artiodactyla, 39%; Carnivora, 26%; Primates, 24%; Diprotodontia, 8%; Lagomorpha, 3%) and 19 species (see Figs. 1 and 2 and Additional file 1: S1 and Additional file 2: Appendicies for the full dataset), consisting mostly of domestic dogs (12 studies, 138 effect sizes), humans (14 studies, 76 effect sizes), bare-nosed wombats (4 studies, 67 effect sizes) and Iberian ibex (8 studies, 63 effect sizes).

#### Type I hypersensitivity immune variable changes

Of the parameters examined for their association with host disease status, all leukocyte types exhibited a significant change (increase or decrease with CIs not overlapping zero) in one or more of the mange status and severity categories, except monocytes and basophils (Table 1). These changes included 3 species and 10 individual studies. Overall leukocyte and lymphocyte numbers were only significantly elevated in the moderate and severe mange categories, respectively. However, significant effect size changes in the numbers of several other cell types were observed across the spectrum of disease severity. For variables where increases were observed from mild to severe disease (mast cells, neutrophils and macrophages), effect sizes also increased sequentially, except from eosinophils, where the effect size peaked at moderate mange severity. T helper CD4+ were the only leukocyte type that decreased in effect size. The only antibody or cytokine with measurements reported in studies with a mange severity scale was IgE, which showed an increase in effect sizes with severity. Antibodies IgG and IgM, and cytokines IL-2, IL-4, IL-5, IL-10, IL-13, and transforming growth factor (TGF)-ß were elevated in diseased relative to control comparisons. Effect sizes for monocytes, basophils, monocyte chemoattractant protein (MCP)-1, macrophage inflammatory protein (MIP)-1ɑ, IgA, IL-8, IL-17, tumor necrosis factor (TNF)-α, interferon (IFN)-γ and complement (C) 3 were not significantly changed in relation to disease status.

**Table 1:** Effect sizes and 95% confidence intervals (round parentheses) for immunological parameters measured during sarcoptic mange in hosts exhibiting Type I hypersensitivity response

Response	Ctrl vs affected	Ctrl vs mild	Ctrl vs moderate	Ctrl vs severe
Total leukocytes	4.12 (− 1.50; 9.75) [8]	0.75 (− 0.22; 1.72) [2]	0.31 (− 0.76; 1.39) [1]	2.74 (**1.33; 4.14**) [1]
Lymphocytes	0.18 (− 1.24; 1.61) [8]	0.48 (− 3.12; 4.10) [2]	16.13 (**11.99; 20.28**) [1]	− 0.02 (− 6.96; 6.91) [2]
Eosinophils	3.84 (**0.63; 7.05**) [8]	2.48 (**1.50; 3.47**) [2]	3.84 (**2.64; 5.04**) [1]	2.44 (**0.88; 3.99**) [2]
Mast cells	–	2.28 (**1.37; 3.19**) [1]	2.79 (**1.79; 3.79**) [1]	4.18 (**2.91; 5.46**) [1]
Neutrophils	3.58 (− 1.01; 8.17) [8]	1.53 (**0.89; 2.17**) [2]	1.63 (**0.81; 2.45**) [1]	3.01 (**2.16; 3.86**) [2]
Monocytes	− 0.14 (− 1.03; 0.75) [7]	− 0.08 (− 1.01; 0.83) [1]*		− 0.07 (− 1.07; 0.93) [1]
Basophils	0.41 (− 0.78; 1.61) [3]	–	–	–
Macrophage	–	1.58 (**0.77; 2.40**) [1]	2.31 (**1.39; 3.23**) [1]	8.17 (**5.98; 10.35**) [1]
CD4+	− 0.68 (**− 1.36; − 0.001**) [2]	–	–	–
MCP-1	0.35 (− 0.03; 0.73) [1]	–	–	–
MIP-1a	0.05 (− 0.32; 0.44) [1]	–	–	–
IgE	12.57 (− 8.26; 33.41) [5]	0.98 (**0.72; 1.23**) [2]	2.43 (**2.01; 2.85**) [2]	5.10 (**0.08; 10.13**) [2]
IgG	2.01 (**1.10; 2.92**) [2]	–	–	–
IgA	− 0.65 (− 1.96; 0.65) [2]	–	–	–
IgM	1.46 (**0.98; 1.93**) [2]	–	–	–
IL-2	2.39 (**1.13; 3.65**) [1]	–	–	–
IL-4	5.04 (**0.05; 10.04**) [3]	–	–	–
IL-5	7.69 (**3.80; 11.59**) [3]	–	–	–
IL-8	43.80 (− 42.10; 129.71) [2]	–	–	–
IL-10	2.36 (**1.67; 3.05**) [1]	–	–	–
IL-13	3.39 (**2.83; 3.95**) [1]	–	–	–
IL-17	0.05 (− 0.33; 0.44) [1]	–	–	–
TNF-α	− 7.26 (− 22.10; 7.57) [2]	–	–	–
TGF-β	9.08 (**5.95; 12.20**) [1]	–	–	–
IFN-γ	− 0.02 (− 0.41; 0.35) [1]	–	–	–
C3	− 1.98 (− 4.96; 0.99) [2]	–	–	–

The number of parameters across studies are provided in square parentheses. A dash represents that no data was available in the category for the given parameter. References for each parameter can be found in Additional file 2: S2

Significant effect sizes (confidence intervals not overlapping zero) are highlighted in bold

Ctrl, control; MCP-1, Monocyte Chemoattractant Protein-1; MIP-1a, Macrophage Inflammatory Protein-1; IgE, Immunoglobulin E; IgG, Immunoglobulin G; IgA, Immunoglobulin A; IgM, Immunoglobulin M; IL-2, interleukin-2; IL-4, interleukin-4; IL-5, interleukin-5; IL-8, interleukin-8; IL-10, interleukin-10; IL-13, interleukin-13; IL-17, interleukin-17; TNF-α, tumour necrosis factor alpha; TGF-β, Transforming growth factor beta; IFN-γ, Interferon-gamma; C3, Complement 3

^*^mild/moderate mange disease grouped together in the original article

#### Type IV hypersensitivity immune variable responses

Fewer immune variable measurements were investigated in association with Type IV hypersensitivity responses to *S. scabiei* infection (Table 2, 20 for Type IV vs. 26 for Type I). Although severity data was available for total leukocytes, lymphocytes, neutrophils, eosinophils, monocytes and mast cells, significant increases in effect sizes were only observed in monocytes in mild and severe cases, in neutrophils and eosinophils in mild cases of mange, as well as in total leukocytes and monocytes in affected cases relative to controls. Although no significant changes were observed in lymphocyte number, T cells, CD4+, and CD8+ cells were all significantly elevated in diseased relative to control comparisons. Macrophage numbers were not measured directly in hosts exhibiting Type IV hypersensitivity response, however, MCP-1, a key chemokine for regulation of macrophage migration and infiltration, increased significantly in diseased relative to control comparisons. Of the antibodies, only IgG have been examined in relation to mange severity categories, which showed an increase in severe cases, as well as in diseased relative to control comparisons. Antibody IgM increased significantly, whereas the effect size of IgA decreased significantly (both in diseased relative to control comparisions). The only significant change in cytokines was observed in IL-17 that increased in diseased relative to control comparisons. Changes in lymphocytes, basophils, mast cells, IgE, TNF-a, IL-1, IL-6, and IL-8 did not significantly differ relative to infection status or disease severity.

**Table 2:** Effect sizes and 95% confidence intervals (round parentheses) for immunological parameters measured during sarcoptic mange in hosts exhibiting Type IV hypersensitivity response

Response	Ctrl vs affected	Ctrl vs mild	Ctrl vs moderate	Ctrl vs severe
Leukocytes	0.63 (**0.03; 1.23**) [6]	1.16 (− 0.11; 2.44) [3]	1.03 (− 1.14; 3.21) [2]	4.14 (− 0.28; 8.56) [3]
Neutrophils	0.71 (− 0.04; 1.47) [11]	0.78 (**0.04; 1.52**) [4]	0.39 (− 0.38; 1.16) [2]	2.89 (− 0.91; 6.70) [4]
Lymphocytes	− 0.21 (− 1.34; 0.92) [8]	0.41 (− 0.01; 0.83) [4]	0.14 (− 0.24; 0.52) [3]	0.96 (− 0.49; 2.42) [4]
T cells	1.32 (**0.01; 2.62**) [1]	–	–	–
Monocytes	0.81 (**0.20; 1.42**) [7]	0.41 (**0.05; 0.76**) [2]	− 0.07 (− 0.51; 0.66) [1]	1.76 (**1.34; 2.18)** [2]
Basophils	0.37 (− 0.47; 1.22) [4]	–	–	–
Eosinophils	1.16 (− 0.74; 3.07) [8]	0.76 (**0.42; 1.10**) [3]	0.42 (− 0.03; 0.88) [2]	0.88 (− 0.83; 2.60) [3]
Mast cells	–	0.71 (− 1.65; 0.21) [1]	0.44 (− 1.16; 0.27) [1]	0.43 (− 1.12; 0.26) [1]
CD4+	5.41 (**4.51; 6.31**) [1]	–	–	–
CD8+	5.23 (**4.35; 6.11**) [1]	–	–	–
MCP-1	5.58 (**4.65; 6.52**) [1]	–	–	–
IgE	5.04 (− 4.27; 14.35) [2]	–	–	–
IgG	1.33 (**0.34; 2.33**) [4]	1.73 (− 0.54; 4.00) [3]*		2.81 (**0.35; 5.27**) [3]
IgA	− 0.63 **(− 1.17; − 0.09**) [1]	–	–	–
IgM	1.46 (**0.87; 2.05**) [1]	–	–	–
IL-1	0.00 (− 1.08; 1.08) [5]	–	–	–
IL-6	1.25 (− 2.04; 4.55) [3]	–	–	–
IL-8	− 0.98 (− 2.23; 0.27) [3]	–	–	–
IL-17	27.65 (**23.54; 31.76**) [1]	–	–	–
TNF-α	0.61 (− 0.70; 1.93) [2]	–	–	

The number of parameters across studies are provided in square parentheses. A dash represents that no data was available in the category for the given parameter. References for each parameter can be found in Additional file 2: S2

Significant effect sizes (confidence intervals not overlapping zero) are highlighted in bold

Ctrl, control; MCP-1, Monocyte Chemoattractant Protein-1; MMI, macrophage migration inhibitor test; TNF-α, Tumour Necrosis Factor alpha; IL-1, Interleukin-1; IL-5, Interleukin-5; IL-6, Interleukin-6; IL-8, Interleukin-8; IL-17, Interleukin-17; IgG, Immunoglobulin G; IgE, Immunoglobulin E; IgM, Immunoglobulin M; IgA, Immunoglobulin A

^*^mild/moderate mange disease grouped together in the original article

#### Oxidant/antioxidant status

All but one of the parameters associated with oxidative stress, mentioned in the literature, exhibited a significant change (increase or decrease with CIs not overlapping zero) (Table 3). Lipid peroxidation (LPO), free gluthathione (GSH), total oxidative stress (TOS) and malondialdehyde (MDA) had elevated effect sizes relative to control and/or increased with disease severity. Catalase (CAT), vitamin C, zinc, and copper were reduced, relative to the controls and/or with disease severity. Free glutathione:oxidised glutathione ratio (GSH:GSSH) decreased significantly in severe mange cases, but no significant change was observed in diseased relative to control comparisons nor in mild or moderate mange severity. Superoxide dismutase (SOD) did not significantly differ relative to infection status or disease severity.

**Table 3:** Effect sizes and 95% confidence interval (CI) for oxidant/antioxidant, acute phase protein, erythrocytic, hepatological and nephrological variables measured for hosts with mange (Type I and IV hypersensitivity)

Response	Ctrl vs affected	Ctrl vs mild	Ctrl vs moderate	Ctrl vs severe
Oxidant/antioxidant status				
LPO	5.68 (**0.34; 11.02**) [4]	2.36 (**0.01; 4.72**) [3]	4.23 (− 0.52; 8.99) [2]	3.05 (**1.57; 4.54**) [1]
SOD	− 4.22 (− 8.99; 0.55) [4]	− 5.82 (− 14.95; 3.31) [7]	− 12.43 (− 31.24; 6.37) [6]	− 1.44 (− 31.24; 6.37) [4]
CAT	− 2.39 (− 6.58; 1.79) [3]	− 1.34 (− 3.73; 1.05) [6]	− 2.38 (**− 4.58; − 0.17**) [5]	− 2.48 (**− 4.252; − 0.71**) [4]
GSH	− 5.46 (**− 10.59; − 0.34**) [6]	− 6.56 (− 18.07; 4.94) [5]	− 4.71 (− 9.92; 0.48) [5]	− 3.56 (− 8.35; 1.22) [4]
GSH:GSSH	–	0.10 (− 0.54; 0.75) [1]	− 0.27 (− 0.93; 0.37) [1]	− 0.83 (**− 1.50; − 0.15**) [1]
TOS	2.54 (**1.59; 3.49**) [1]	0.02 (− 0.62; 0.67) [1]	0.18 (− 0.46; 0.83) [1]	1.64 (**0.90; 2.38**) [1]
MDA	− 0.12 (− 0.73; 0.48) [1]	0.41 (− 0.15; 0.97) [2]	1.10 (**0.42; 1.77**) [2]	1.66 (**0.46; 2.87**) [1]
Zinc	− 1.03 (**− 1.87; − 0.19**) [4]	− 1.79 (− 5.37; 1.77) [2]	− 0.73 (**− 1.35; − 0.10**) [1]	− 4.14 (**− 5.93; − 2.35**) [1]
Copper	− 0.47 (**− 0.86; − 0.08**) [4]	− 1.03 (− 2.29; 0.23) [4]	− 3.90 (− 10.13; 2.32) [4]	− 3.12 (**− 4.62; − 1.61**) [1]
Vitamin C	− 3.52 (**− 5.14; − 1.91**) [5]	− 1.58 (− 3.36; 0.19) [3]	− 9.72 (− 26.06; 6.60) [2]	− 13.23 (− 35.14; 8.68) [3]
Acute phase protein response				
Ceruloplasmin	1.35 (**0.71; 1.99**) [1]	–	–	–
Haptoglobin	0.69 (− 0.07; 1.45) [3]	− 0.36 (− 0.78; 0.05) [1]*		0.52 (− 0.13; 1.18) [1]
Transferrin	1.87 (**1.20; 2.53**) [1]	–	–	–
AGP	0.53 (**0.16; 0.90**) [1]	0.60 (**0.18; 1.01**) [1]*		1.14 (**0.52; 1.76**) [1]
SAA	68.14 (− 64.55; 200.84) [2]	0.77 (− 0.68; 2.23) [2]	0.64 (− 0.07; 1.36) [1]	1.89 (**0.95; 2.83**) [1]
Albumin	− 0.73 (**− 1.16; − 0.30**) [14]	0.45 (− 1.14; 2.05) [7]	− 1.04 (**− 1.87; − 0.21**) [5]	− 3.38 **(− 6.68; − 0.07**) [6]
Erythrocyte changes				
Haemoglobin	− 0.86 (**− 1.31; − 0.41**) [17]	− 3.07 (− 7.07; 0.92) [6]	− 3.67 (− 8.37; 1.02) [4]	− 3.81 (**− 6.74; − 0**.**88**) [5]
RBC	− 1.32 (**− 2.00; − 0.64**) [13]	− 0.71 (**− 1.06; − 0.36**) [7]	− 1.48 (**− 2.11; − 0.85**) [4]	− 2.42 (**− 3.71; − 1.11**) [5]
Haematocrit	− 1.70 (**− 2.99; − 0.40**) [16]	− 1.98 (**− 3.26; − 0.70**) [6]	− 3.02 (**− 5.99; − 0.04**) [4]	− 4.52 (− 9.24; 0.18) [5]
MCV	− 0.27 (− 1.16; 0.61) [5]	− 0.82 (**− 1.45; − 0.18**) [3]	− 0.07 (− 0.46; 0.31) [3]	1.51 (**0.83; 2.19**) [2]
MCH	0.17 (− 0.30; 0.65) [3]	− 0.23 (− 1.91; 1.44) [2]	− 0.50 (− 1.78; 0.77) [2]	− 0.06 (− 0.97; 0.85) [1]
MCHC	− 0.11 (− 0.59; 0.36) [3]	− 0.53 (− 4.05; 2.97) [2]	− 0.64 (− 2.08; 0.85) [2]	− 1.51 (**− 2.46; − 0.55**) [1]
Iron	− 1.10 (**− 2.03; − 0.16**) [5]	− 1.23 (− 5.20; 2.74) [2]	0.44 (− 0.64; 1.53) [1]	− 3.69 (**− 5.35; − 2.03**) [1]
Hepatological and nephrological changes				
Total protein	− 0.80 (− 1.88; 0.27) [16]	− 0.22 (− 0.51; 0.07) [5]	− 0.33 (− 0.92; 0.26) [3]	− 0.76 (− 1.99; 0.47) [4]
Globulin	0.11 (− 0.57; 0.80) [20]	− 0.17 (− 1.03; 0.67) [1]	0 (− 1.04; 1.04) [1]	− 0.10 (− 1.15; 0.93) [1]
ALT	1.68 (**0.45; 2.90**) [6]	2.21 (− 2.75; 7.18) [4]	7.53 (− 7.48; 22.54) [2]	7.31 (− 6.03; 20.65) [4]
GGT	–	2.95 (− 3.21; 9.11) [2]	11.29 (− 12.33; 34.92) [2]	29.02 (**18.58; 39.45**) [1]
Bilirubin	0.79 (− 0.12; 1.72) [6]	0.21 (− 0.40; 0.84) [2]*		1.62 (− 3.50; 6.75) [2]
BUN	2.06 (− 0.20; 4.32) [4]	0.16 (− 0.99; 1.31) [3]	1.33 (**0.36; 2.31**) [2]	0.83 (**0.41; 1.25**) [1]
Creatinine	1.13 (− 0.98; 3.26) [8]	− 2.57 (− 5.81; 0.65) [6]	− 2.15 (− 5.44; 1.13) [5]	− 4.08 (− 12.17; 4.01) [4]
BUN:Creatinine	–	1.09 (− 0.03; 2.22) [1]	1.87 (**0.64; 3.10**) [1]*	
Urea	0.33 (− 1.59; 2.25) [4]	− 2.42 (− 5.19; 0.35) [2]	− 11.51 (− 35.59; 12.56) [2]	− 3.07 (− 11.49; 5.34) [2]
Urea:Nitrogen	–	–	− 0.59 (− 1.33; 0.14) [1]	0.70 (**0.01; 1.38**) [1]

The number of parameters across studies are provided in square parentheses. A dash represents that no data was available in the category for the given parameter. References for each parameter can be found in Additional file 2: Appendix

Significant effect sizes (confidence intervals not overlapping zero) are highlighted in bold

Ctrl, control; LPO, Lipid peroxidation; SOD, Superoxide dismutase; CAT, Catalase; GSH, Free glutathione; GSH:GSSH, Free glutathione:oxidized glutathione ratio; TOS, Total oxidative stress; MDA, Malondialdehyde; AGP, Acid(1)-alpha glycoprotein; SAA, Serum amyloid A; RBC, Red blood cell; MCV, Mean corpuscular volume; MCH, Mean corpuscular haemoglobin; MCHC, Mean corpuscular haemoglobin concentration; ALT, Alanine aminotransferase; GGT, Gamma-glutamyl transferase; BUN, Blood urea nitrogen

^*^mild/moderate mange disease grouped together in the original article

#### Acute phase protein response

Three out of the six acute phase proteins (APPs) mentioned in the literature showed increased effect sizes in diseased relative to control comparisons [e.g. ceruloplasmin, transferrin, acid(1)-alpha glycoprotein (AGP)]. Serum amyloid A (SAA) increased in severe cases, but not in mild and moderate disease categories and in diseased relative to control comparisons. Only albumin of the APPs decreased in effect size in both moderate and severe cases of infection and in diseased relative to control comparisons. Haptoglobin was the only acute phase protein in the analysis without evidence of any significant changes in either disease categories.

#### Erythrocytic changes

Six out of seven erythrocytic parameters, mentioned in literature, significantly decreased in one or more of the mange severity categories (Table 3). Total red blood cells (RBC) decreased significantly in all categories. Haemoglobin and iron decreased in severe cases of mange and in diseased relative to controls. Haematocrit values decreased in mild and moderate cases of mange and in diseased relative to controls. Mean corpuscular haemoglobin concentration (MCHC)’s effect size decreased in severe cases of mange. In contrast, the mean corpuscular volume (MCV) had an elevated effect size in severe cases of mange, compared to controls. Mean corpuscular haemoglobin (MCH) was the only parameter not showing any significant changes in effect sizes.

#### Hepatological and nephrological changes

Only five out of ten parameters, mentioned in the literature, had significant effect size changes. Alanine aminotransferase (ALT), increased in diseased relative to control comparisons. Gamma-glutamyl transferase (GGT) significantly increased in severe cases. Blood urea nitrogen (BUN) was increased in moderate and severe cases of sarcoptic mange compared to controls (no data for severe cases of mange). The BUN:creatinine ratio had a significant increased effect size in moderate/severe cases, but not in mild. There was no data in diseased relative to control comparisons for the BUN:creatinine ratio. Urea:Nitrogen ratio increased significantly in severe cases as well, but not in moderate. No data was available for mild cases of disease nor diseased relative to control comparisons for Urea:N ratio. The remaining parameters (total protein, globulin, bilirubin, creatinine and urea) did not significantly change with *S. scabiei* infection status or severity.

## Discussion

Sarcoptic mange affects a large range of mammal species globally, with clinical manifestations of disease generally depending on the type of hypersensitiy response experienced by the host (Type I and IV). Thus, combining studies across host species with a meta-analysis provides a fuller understanding of the clinical pathology of sarcoptic mange. We brought together both narrative reviews on the topic, which help develop proposed immunological processes/hypotheses, and empirical studies, formally examined with the use of a meta-analysis. We showed which immunological parameters changed significantly during Type I and Type IV hypersensitivity responses, as well as oxidant/antioxidant balance, APPs, erythrocytic, hepatological and nephrological changes compared to controls. Overall, our results reveal the complex nature of immune and clinical pathological changes associated with scabies/mange. As anticipated, our empirical findings broadly align with narrative reviews (see Fig. 1), although they also simplify proposed processes. Our results provide evidence of interspecific consensus in 27 immunological and clinical pathology variables across Type I (6/26 parameters) and Type IV (3/20 parameters) hypersensitivity, and in oxidant/antioxidant balance (6/10 parameters), APPs (3/6 parameters), erythrocytic (4/7 parameters) and hepatological/nephrological (5/10 parameters) changes. The only parameter showing interspecific consensus in both Type I and Type IV hypersensitivity responses was neutrophils, which showed a similar increase in effect sizes across severity and in affected compared to controls.

### Type I hypersensitivity-associated changes

The immediate immune response associated with Type I hypersensitivity reactions is considered to be primarily driven by a combination of IgE, mast cells and eosinophils [53]. Activation of these immunological variables are confirmed in our meta-analysis. Eosinophils, which primarily help promote inflammation in response to a parasitic infection, increased in scabies/mange affected hosts relative to controls and across disease severity. IgE stimulates mast cells and basophils to secrete proinflammatory reagents in response to infection [54–56]. Our results indicated increased levels of IgE and mast cells with mange severity, which corresponds to what is described in the literature [57, 58]. There was evidence of interspecific consensus in the response of eosinophils and IgE, including humans, domestic dogs and pigs, and humans, bulls, rabbits and domestic dogs, respectively.

Looking at the effect sizes of changes in lymphocyte numbers, an increase was observed in moderate cases compared to controls, but not in other severity categories. Importantly, results in this moderate severity category only stem from a single study involving domestic dogs. Increased lymphocytes numbers could be due to the increased pruritus and the subsequent abrasion of the skin, which enhances microbial invasion leading to the increase of white blood cells [29]. However, both intra- and inter-specific variation was evident in mild and severe cases of mange with both non-significant decreases and increases in effect sizes being documented. This suggests variation when it comes to lymphocyte numbers in response to *S. scabiei* infection. A potential explaination for this variation could be linked to the shift or overlap between Th1 and Th2 responses [59], discussed further below.

According to narrative reviews (illustrated in Fig. 1A) of Type I hypersensitivity response to *S. scabiei* infection affected hosts most often exhibit a predominant Th1 response. With regards to specific lymphocytes lineages, our meta-analysis provides some evidence of significant changes in response to disease. For example, a single study showed significant decreases in CD4+ T cells in diseased domestic dogs relative to controls indicating cell differentiation from naïve CD4+ T cells to specific linages such as Th1 and/or Th2 T cells [60]. Contrastingly, we observed significant increases in cytokines that are linked to an upregulation of the Th1 response, such as IL-2 in dogs, as well as the Th2 response, such as IL-4 in dogs and humans [59, 61, 62]. We also observed increases in effect sizes for IL-5 in dogs and humans, and IL-10 and IL-13 in dogs, which have all been linked to both Th1 and Th2 cells in humans [61]. While there remains uncertainty about the precise role of these cytokines in non-human mammals, the interspecific consensus between humans and dogs with regards to IL-4 and IL-5 could indicate a more intricate immune response with an overlap between Th1/Th2 response in sarcoptic mange affected hosts, or a shift from one response to another depending disease severity. Another surprising result was the fact that we did not observe a significant change or increase in the effect size for IFN-y in a study involving humans which is linked to a Th1 response in humans [59], perhaps providing further evidence of more mixed response. In order to fully understand the complex processes occurring, further research into the associated lymphocytes lineages and relevant cytokines within and across host species is warranted.

The meta-analysis also provided evidence of other changes among white blood cell types and immunoglobulins. Overall leukocyte numbers increased significantly in severe cases of mange compared to controls, however this was derived from a single dog study. Looking at the diseased relative to control category, the overall effect size was not significant but not surprisingly all eight studies (human n = 5, dog n = 3) showed a general increase indicating interspecific consensus of leukocyte numbers. In the remaining individual white blood cell types we observed a significant increase in effect sizes of neutrophils. Effect sizes from two studies (human and domestic dog) measuring disease severity showed an increase of neutrophils, but the relationship was less clear for the infected vs. control (n = 8). Looking at the individual studies in the diseased relative to controls, neutrophils in 6/8 studies significantly increased (n = 3 dog studies, n = 3 human studies), and two studies did not (one pig study, one dog study). This could indicate a more varied neutrophil response depending on host, however it could also depend on disease severity of the infected hosts in the diseased relative to controls category.

Only one study (dog) of hosts affected with sarcoptic mange measured the change in macrophages. In the study we observed a significant increase in the effect size in mild, moderate and severe cases, as well as with increased severity, consistent in other infections causing chronic inflammation [63, 64]. Macrophage levels or activation is linked with the host’s innate immune response in regards to the Th1/Th2 pathway activation [65]. The cytokine TGF-ß increased in diseased relative to controls (from a single study in dogs). TGF-ß plays an important role in many regulatory functions, such as wound healing and immunoregulation. The increase observed in dogs could be linked to its chemoattractant properties for macrophages, as well as other key cells at the site of inflammation [66]. In some instances IgG antibody production might increase and take over from IgE antibodies in high infestations, as seen in *R. microplus* studies in cattle [67]. This could be the reason why we observed a significant increase in IgG effect size in diseased relative to control comparisons (n = 2, both humans). Since we do not have any information regarding changes with disease severity we can only speculate. IgM, associated with the immediate immune response to microbial infection [68] and the activation of the complement system [69], increased significantly in diseased relative to controls (n = 2, both humans). Again we do not have any information with disease severity.

### Type IV hypersensitivity-associated changes

Type IV hypersensitivity is characterised by the activation of T cells by antigen-presenting cells, such as macrophages and dentritic cells. This promotes cytokine secretion [53] by T cells as well as T cell differentiation into helper T cell CD4+ and cytotoxic T cell CD8+, which further promotes cytokine secretion and recruits other white blood cell types such as neutrophils [70]. This is somewhat evident in our meta-analysis results, however there were generally fewer parameters available for analysis in host exhibiting Type IV hypersensitivity than Type I hypersensitivity response, which limits interpretation.

Total T cells significantly increased in a single bare-nosed wombat study. T cells can futher be divded into two types; the helper T cells and cytotoxic T cells, which we have data on in one human study (significantly increased effect sizes). The activation of macrophages is a bit harder to quantify, as we do not have a direct measure of macrophage levels in hosts exhibiting Type IV hypersensitivity available. However, MCP-1, a key chemokine for regulation of migration and infiltration of monocytes, memory T lymphocytes and natural killer cells [71], from a human study suggested a potential increase in monocytes/macrophages at the inflammation site. This is further suggested by an increase in monocytes in diseased relative to control comparisons (n = 7, 5 animal species), as well as in mild and severe cases (n = 2, Iberian ibex, raccoon dog).

No change in lymphocytes was observed (diseased relative to controls, n = 8; severity range, n = 4). Looking at the individual studies the explaination for the non-significant effect sizes is probably due to interspecific variation. In disease relative to control comparison studies two studies (both BNW) decreased significantly, three studies (goat, san joaquin kit fox, southern hairy-nosed wombat) increased, and the last three studies (Iberian ibex, camel, BNW) non-significantly decreased. The same pattern is evident in studies across severity with species including raccoon dog, Iberian ibex and chamois. Another suprising result is that we only observe a significant increased effect size in neutrophils in mild cases of infection (n = 2, raccoon dogs and Iberian ibex). In both moderate and severe cases, as well as in diseased relative to control comparisons we observe a mixture of increased and decreased effect sizes indicating interspecies variation.

According to narrative review papers (Additional file 1: S1), it is most common to observe a non-protective Th2 dominant response in crusted scabies/mange [13, 72]. However, it is important to note that reviews describing this occurrence have been mostly human focused with a few exceptions (red fox, chamois, ibex and Scandinavian canids, Additional file 1: S1). A review focused on scabies in humans, argued that IL-1 and IL-6 promoted the secretion of IL-17 by T cells, and the increased expression of these cytokines could be contributing to the inflammation observed in crusted scabies patients [73]. IL-8 has been found to be secreted during infestation of *S. scabiei* to promote the recruitment of neutrophils to the site of infection. Only IL-17 of these exhibited significant changes in our meta-analysis, and this was in a single study on humans. IL-1 and IL-6 did not significantly differ and neither did IL-8. This could be due to the nature of the studies involved in these results. To better understand if IL-1, IL-6 and IL-8 change in hosts affected by sarcoptic mange in situ (as oppose to in vitro) experimental studies would be valuable.

For antibody studies, we detected increased effect size in IgG in both diseased relative to control comparisons (n = 4) and in severe cases (n = 3). Species covered in these results are chamois, Iberian ibex and human indicating interspecific consensus. In single species studies we also observed a decrease in IgA (Table 2) linked to the Th2 response in type IV hypersensitivity, and an increase in IgM.

In general, for Type IV hypersensitivity, we observed an overrepresentation of human only studies (e.g., all cytokines, interleukines and antibodies, except for IgG). We also observed many effect sizes could only be calculated for single studies (e.g., CD4+, CD8+, IgA, IgM, IL-17 and MCP-1). Expansion of immune investigations association with Type IV hypersensitivity to other host species would be beneficial for a general understanding of Type IV immunological response to *S. scabiei*. The variable results in Table 2 might also be due to the range of severity that can occur during type IV hypersensitivity responses.

### Oxidative/antioxidant balance

The oxidative/antioxidant balance impacts the progression of inflammatory diseases including sarcoptic mange. If antioxidant defenses during inflammation are insufficient or inadequate it can lead to cell death and damage due to excessive amount of free radicals/reactive species [74], shifting the balance towards potential oxidative stress and if left unchecked exacterbation of disease development [75]. Oxidant/antioxidant balance during sarcoptic mange has been investigated in several species, such as dogs, pigs, water buffalo, camels, and humans. We found evidence of an altered antioxidant defence in hosts affected by sarcoptic mange, evidenced by increased levels of lipid peroxidation (LPO), total oxidative stress (TOS) and malondialdehyde (MDA), as well as decreased levels of catalase (CAT). Vitamin C, an antioxidant [30], also decreased, in diseased relative to control comparisons, suggesting increased free radicals leading to further oxidative stress. This is supported by the decrease of trace minerals such as zinc in moderate and severe cases of sarcoptic mange, as well as copper which is linked to both the acute phase protein ceruloplasmin and the body’s antioxidant defence in combination with zinc and superoxide dismutase (Cu–Zn-SOD) [76]. The decreased effect size of vitamin C in combination with decreased levels of free glutathione (GSH) could also indicate potential sepsis in mange affected hosts, as observed in critically ill patients [74, 77]. All of the significant parameters covered several species each, suggesting interspecific consensus among these.

Surprisingly, superoxide dismutase (SOD) did not provide any significant results, both in diseased relative to control and across severity, despite there being a general consensus of decreased levels of SOD in mange affected hosts (8/10 studies). The general decrease (albeit not significant) does follow the expected trend if the oxidant/antioxidant balance were shifting towards oxidative stress.

### Acute phase proteins

Acute phase proteins (APPs) can have many different functions under infection, inflammation, or stress, which can be species-specific [78]. In response to infection or inflammation the liver produces several APPs while consecutively reducing others, subsequently classifying them into two categories; negative APPs and positive APPs. Negative APPs include albumin and transferrin, whereas positive APPs include serum amyloid A (SAA), ceruloplasmin, haptoglobin and AGP [79]. The increase observed in our results, in all but albumin, come mostly from research on Alpine ibex (*Capra ibex*) [22] and Iberian ibex (*Capra pyrenaica*) [78] with a small contribution of a pig study [44] in transferrin and ceruloplasmin. The increases in effect sizes of ceruloplasmin, AGP and SAA corresponds to what is expected in the host response to infection as illustrated in dogs, pigs and cattle [79]. SAA is secreted during the acute phase of inflammation with a key role of recruiting white blood cell types to the site of inflammation [79]. Surprisingly, we only observed a significant effect size in severe cases of mange in Iberian ibex relative to controls. This is particularly surprising as we would expect APPs to be highest in early stages of infection (mild and moderate cases) and slowly wane with chronic disease (e.g. severe cases) [80], however chronic expression of SAA has been observed in rats causing systemic amyloidosis [81] indicating that it is likely species and/or condition specific APP responses. Another unexpected result is the lack of significance in haptoglobin. Haptoglobin is known to increase several fold during an inflammatory event and the levels (e.g. high or low of haptoglobin subtypes) of haptoglobin has an important role in the disease development for other parasitic diseases in regards to exacterbated oxidative stress [82]. Looking at the individual studies’ effect sizes the two studies with Type I hypersensitivity response (pig and alpine ibex) have significantly increased effect sizes, whereas the Iberian ibex study (involving both severity range and diseased relative to control) exhibiting Type IV hypersensitivity had non-significantly increased effect sizes. To understand if this difference is due to Type I or Type IV hypersensitivity responses or due to species variation or lastly a function of the assays used in the analysis, more research is needed.

Albumin is the most commonly studied APP in our analysis incompassing 12 different species in total. Despite its antioxidant properties that could influence oxidative status, as a negative acute phase protein its decreased levels are most-likely explained by reduced hepatic synthesis [30] and catabolic processes during sarcoptic mange [35, 83]. Unexpectedly, the effect size of transferrin, in diseased relative to control comparisons, showed an increase. This contradicts other studies into immune-modulated inflammation, as these found that transferrin, which is a negative APP associated with the innate immune response [79], typically decreases during inflammation [84, 85]. Increased levels of transferrin could be due to overload of free iron in the blood which could be indicative of anaemia [86]. In general, more studies and species are needed in regard to acute phase protein responses to obtain a comprehensive understanding the APPs role in sarcoptic mange affected hosts.

### Erythrocytic, hepatological and nephrological changes

The occurance of anaemia can have many underlying causes with for example low levels of blood cells or iron being linked to side effects of prolonged/chronic disease (e.g. anaemia of chronic disease), including increased oxidative stress due to cell death [75]. Anaemia is usually confirmed by decreased levels of total red blood cells (RBC), haemoglobin and haematocrit [87, 88]. Our meta-analysis provided evidence of anaemia in diseased relative to control comparisons in mange affected hosts with decreasing effect sizes in all three parameters covering 10 to 12 animal species respectively, with additional evidence of anaemia in severe cases of mange with significant decreased effect sizes in haemoglobin and RBC levels.

Another factor complementing RBC results were the decreased effect sizes of iron in both diseased relative to controls and in severe cases of disease, as well as increased levels of transferrin as discussed above. Surprisingly haematocrit were only significantly decreased in mild and moderate cases of mange, not in severe. However, breaking down the individual results we do observe a general decrease of haematocrit values in severe cases of mange across all studies and species (n = 5) suggesting aneamia of chronic disease. MCV and MCHC, sometimes used to classify anaemia, exhibited variable relationships. MCHC decreased significantly in severe cases of mange which might be connected with iron-deficiency anaemia [89], but showed no significant results in any other categories. MCV decreased significantly in mild cases, but increased in severe cases of mange with no significant results in diseased relative to controls despite covering more studies (n = 5). These patterns could be due to variability between hosts, as anaemia was a consequence of sarcoptic mange in some hosts [19, 33, 39], but not others [32, 90], independent from the hypersensitivity reaction that the host exhibited. Although with parameters such as haemoglobin, RBC and haematocrit incompassing more studies, development of anaemia in sarcoptic mange affected hosts is a relatively certain conclusion to draw from these results.

Hepatic and renal function cannot be directly or solely quantified using the parameters reported in the studies analysed by this meta-analysis and must be assessed on an individual basis. Instead, with regards to the liver, we are able to provide potential evidence of general hepatocellular damage, evidenced by increased ALT in diseased relative to controls and increased levels of GGT in severe cases, and use proxy measures such as BUN and APPs to infer overall hepatic function. Regarding the latter, the absence of significant decreases in BUN and significant increases in positive APPs may provide a gross, albeit highly insensitive, indication of appropriate liver function. In light of these findings, it is deemed unlikely that the observed decreases in albumin are a result of hepatopathy [83], although the complex physiological relationship of this parameter with multiple body systems hinders accurate interpretation.

While the observed increases in BUN are not suggestive of hepatic dysfunction, in combination with increased BUN:creatinine ratio these changes may indicate a degree of renal compromise among study subjects [91]. However, in the absence of urine analysis and with only 1–2 studies per parameter our ability to interpret these findings is also severely limited. Decreased or altered kidney function in mange is usually described in combination with post-streptoccocal glomerulonephritis (see Fig. 1A) caused by group A streptococci infections associated with secondary infections [92, 93]. Despite the presence of neutrophilia, more specific research into measures of renal function alongside microbiological culture of urine would be required to rigorously investigate the impacts of mange on the kidneys. Additionally, renal parameters (e.g., urinary urea nitrogen and creatinine ratio (UN:C)) together with organ-fat scores could provide indication of starvation or loss of appetite associated with sarcoptic mange [35, 37].

## Conclusion and future directions

Our meta-analysis provides evidence of elevated levels of IgE, eosinophils and mast cells in hosts exhibiting Type I hypersensitivity response, corresponding to what has been described in narrative reviews, as well as a potential switch between or mixed Th1/Th2 response to the infection. In contrast, results from Type IV hypersensitivity response were more variable. Results suggest, that antibody responses are fairly typical of what we expected to find, however the expected cell-mediated response and a Th2 response were less evident. Additionally, a general response to *S. scabiei* infection across all host species included signs of oxidative stress, especially in severe cases of sarcoptic mange and some signs of potential anaemia of chronic disease, as expected (see Table 4 for summary).

**Table 4:** Overarching interpretations of complex immunological and clinical pathology results from this study

Overall	Meta-analysis shows how fragmented research has been for varying immunological and clinical pathology responses (see Figs. 1–2) across host species
Type I responses	Across papers broadly consistent with expectations from narrative reviews, particularly for eosinophil, mast cell and IgE increases. Antibody response increased (e.g. IgE, IgG and IgM)
Type IV responses	Less evidence supporting typical cell-mediated response from narrative reviews. No evidence of lymphocytosis, despite individual increases in lymphocytic cells of differing lineages (CD4+ /CD8+ /T cells). Antibodies IgG and IgM increased; IgA decreased
Other response types	Acute phase proteins increased in levels with mange Indication of anaemia of chronic disease associated with mange. Results not as clear as what is described in narrative reviews (Fig. 1A) Meta-analysis provides possible evidence of association with hepatocellular damage (GGT, ALT), but research is needed to infer whether function is broadly affected. Some inconsistencies between narrative reviews (Fig. 1A) and our meta-analysis (Fig. 1B) is evident (e.g. globulin) Evidence of shifted oxidant/antioxidant balance toward oxidative stress and indication of potential exacterbated disease development, consistent with what is described in narrative reviews (Fig. 1A)
Interspecific consensus	Increased neutrophils (both Type I and Type IV) Increased IgE in Type I hypersensitivity responses Increased IgG and monocytes in Type IV hypersensitivity responses Consistent changes (increased or decreased) of oxidant/antioxidant parameters Consistent changes (increased or decreased) in acute phase proteins Erythrocytic parameters such as haemoglobin, haemotocrit, MCHC, RBCs decreased consistently
Research directions	Appears to be a general need for studies that more routinely compare results against controls rather than in isolation, and include a more standardised suite of variables No data regarding secondary infections across species, important focus for future studies especially in combination with kidney function Research on microbiomes—potential indicator for reduced protection against pathogenic/harmful microorganisms, leading cause for secondary infections

Importantly, a large proportion of the parameters in the meta-analysis were derived from one or two studies, influencing generalisability, making it difficult to determine if the observed results were (i) species-specific, (ii) simply overrepresented by a single species, (iii) due to the experimental setup (e.g., laboratory animals) or (iv) due to the animals available to sample at that time (e.g., wildlife studies). Another factor is the amount of different pathological parameters available. Studies often select which immunological parameters to include, due to several factors (e.g., funding, focus etc.). This might contribute to why it was difficult to draw definitive conclusions with a limited insight into certain parameters. As discussed previously, articles focusing on diseased compared to healthy individuals, did not specify how severely the diseased group were affected by sarcoptic mange. This could explain the variability observed in Table 2 and why we are not seeing more consistent results overall. To help estimate the true pathology in response to sarcoptic mange, a definition of severity could be implemented in future studies. Furthermore, we found that most of our groupings of data had a publication bias. The reason for this bias is unclear, but could be linked to a general abundance of small studies. In part, it could also be due to studies being more likely to report beneficial results with no effect results remaining unpublished limiting the amount of immunological and clinical pathological parameters available.

Combining immunological and clinical pathological information from multiple host species can help us gain valuable insight to the observed disease responses, especially in diseases that affect a wide range of hosts globally [1]. Here, we undertook a detailed assessment of *S. scabiei*. We hope the information brought together help create and generalise therapeutic strategies, as well as help illustrate disease aspects with little to no information.

## Supplementary Information

**Additional file 1.** Narrative reviews.

**Additional file 2.** Full meta-data.

**Additional file 3.** Reference table.

**Additional file 4.** Figure 1 Flowdiagram & Table 1 Severity groupings.

**Additional file 5.** Funnel plots and Egger’s regression results

## Data Availability

All data generated or analysed during this study are included in this published article and its supplementary information files.

## Abbreviations

Cis: 95% Confidence intervals
IL: Interleukin
IFN-γ: Interferon gamma
TNF-α: Tumour necrosis factor alpha
TGF-β: Transforming Growth Factor beta
CD4+: T helper cells
CD8+: Cytotoxic T cells
Ig: Immunoglobulin
C3: Complement 3
MCV: Mean corpuscular volume
TEC: Total erythrocyte concentration
PCV: Packed cell volume
AGP: Acid(1)-alpha glycoprotein
SAA: Serum amyloid A
A:G ratio: Albumin:globulin ratio
ALT: Alanine aminotransferase
BUN: Blood urea nitrogen
MCHC: Mean corpuscular haemoglobin concentration
MCH: Mean corpuscular haemoglobin
LPO: Lipid peroxidation
CAT: Catalase
GSH:GSSH: Free glutathione:oxidized glutathione ratio
GGT: Gamma-glutamyl transferase
