# Supplementary material for: Evidence underscoring immunological and clinical pathological changes associated with Sarcoptes scabiei infection: synthesis and meta-analysis
Source: BMC Infect Dis. 2022 Jul 28;22:658. doi: 10.1186/s12879-022-07635-5 (PMC9335973; doi:10.1186/s12879-022-07635-5)
Supplement: Supplementary file 3 — Additional file 3. Reference table. [file 12879_2022_7635_MOESM3_ESM.docx]

| Immunological and clinical pathological response | Overview | References |
| --- | --- | --- |
| Type I hypersensitivity | Th1 mediated with increased production of cytokines IFN-y, IL-2, TNF-a, and IL-10.  CD4+ T cells, eosinophils, and macrophages at inflammatory site.  Increased levels of antibodies IgE, IgG and IgA | [1] |
| Type IV hypersensitivity | Th2 mediated with increased production of cytokines IL-4, IL-5, IL-13, IL-17, and IL-23.  Decreased production of IL-10. CD8+ T cells, eosinophils, and fewer macrophages at inflammatory site.  Elevated levels of antibodies IgG, IgE and IgA | [1] |
| Oxidant/antioxidant status | Increased oxidative stress.  Increased production of reactive species and H2O2, as well as iron release from proteins.  Elevated levels of LPO and MDA.  Decreased levels of antioxidants, such as SOD, GSH, vitamin C and vitamin E. | [2, 3] |
| Acute phase protein response | IL-1, IL-2 and TNF-a stimulates hepatocytes to secrete APPs.  Max serum conc. is observed within 24-48 h after initiation. Decline coincides with disease recovery.  APP response (APR) can become chronic if there is continuous stimulation - seen as increased serum conc. of APPs albeit lower than acute episodes of infection. | [3, 4] |
| Erythrocytic changes | Haemolysis: increased reticulocytes, leucocytes, bilirubin, and decreased platelets.  Blood loss: decreased total protein, albumin, iron conc., ferritin (if chronic), platelets and increased reticulocytes. | [5] |
| Hepatological changes | Inflammatory liver disease: Increased AST, ALP, GGT and bilirubin. Decreased albumin, cholesterol, glucose, and BUN.  ALT and AST general indicators of hepatocellular injury. Increases in ALP, AST and bilirubin could indicate liver disfunction. Levels of GGT, GLDH, cholinesterase and globulin are used to complement the beforementioned parameters. Markers for chronic liver diseases are general increase of cytokine levels, such as TNF-a.  Fatty liver disease markers could be glucose levels, triglycerides, and cholesterol levels. | [3, 6] |
| Nephrological changes | Prerenal disease: Normal-increased creatinine, increased phosphorus (severe disease), serum proteins, haematocrit.  Renal disease (e.g. glomerulonephritis): Increased creatinine, phosphorus and urine protein:creatinine ratio, decreased albumin;  Postrenal disease: Increased creatinine and potassium (severe disease) with Indication of blood in urine. | [7] |

1. Bhat, S.A., et al., *Host immune responses to the itch mite, Sarcoptes scabiei, in humans.* Parasit Vectors, 2017. **10**(1): p. 385.

2. Forman, H.J. and H. Zhang, *Targeting oxidative stress in disease: promise and limitations of antioxidant therapy.* Nature Reviews Drug Discovery, 2021.

3. Salem, N.Y., et al., *Canine demodicosis: Hematological and biochemical alterations.* January-2020, 2020. **13**(1): p. 68-72.

4. Jain, S., V. Gautam, and S. Naseem, *Acute-phase proteins: As diagnostic tool.* Journal of pharmacy & bioallied sciences, 2011. **3**(1): p. 118-127.

5. Laboratories, I. *Hematocrit Interpretive Summary*. 2013 11/1/2013 [cited 2021 26 March]; Available from: <https://www.idexx.dk/files/8947-us-hct-interpretive-summary.pdf>.

6. Laboratories, I. *Albumin Interpretive Summary*. 2013 11/1/2013 [cited 2021 16/4]; Available from: <https://www.idexx.no/files/8088-us-albumin-interpretive-summary.pdf>.

7. Laboratories, I., *Blood Urea Nitrogen (BUN) Interpretive Summary.* 2013.
