# Supplementary material for: Evidence underscoring immunological and clinical pathological changes associated with Sarcoptes scabiei infection: synthesis and meta-analysis
Source: BMC Infect Dis. 2022 Jul 28;22:658. doi: 10.1186/s12879-022-07635-5 (PMC9335973; doi:10.1186/s12879-022-07635-5)

Figure 1. Flow diagram of work pipeline


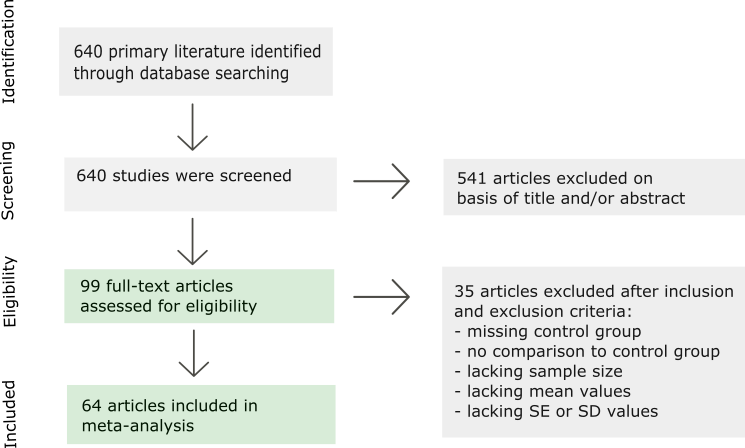


##

Table 1. Classification of sarcoptic mange severity for the purpose of the meta-analysis

| Mange severity | Description |
| --- | --- |
| Control | Healthy |
| Mild | <25% of epidermal surface affected |
| Moderate | 25-50% of epidermal surface affected |
| Severe | >50% of epidermal surface affected |

Figure 2. Krona chart of study distribution of full dataset


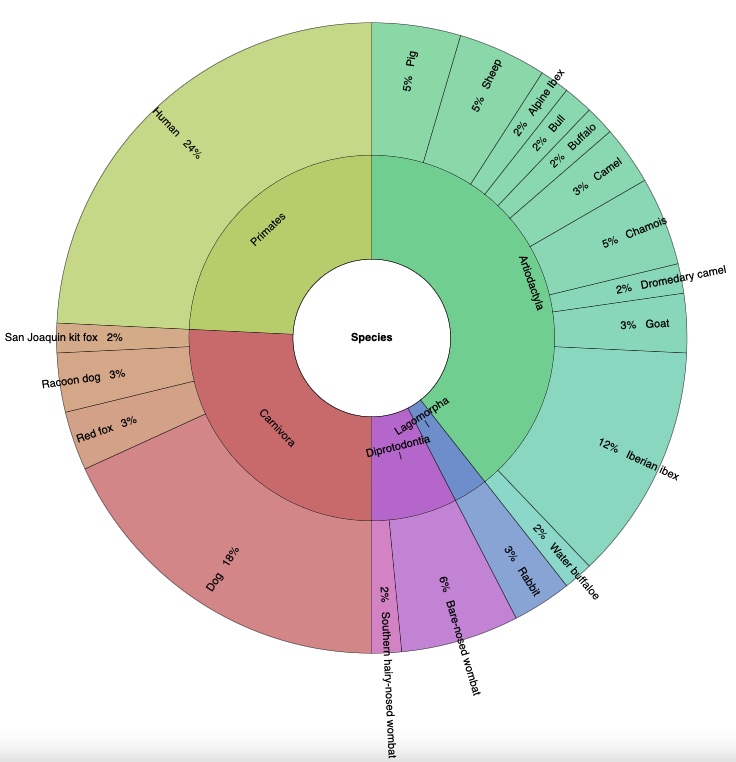


Figure 3. Krona chart of study distribution in type I hypersensitivity response


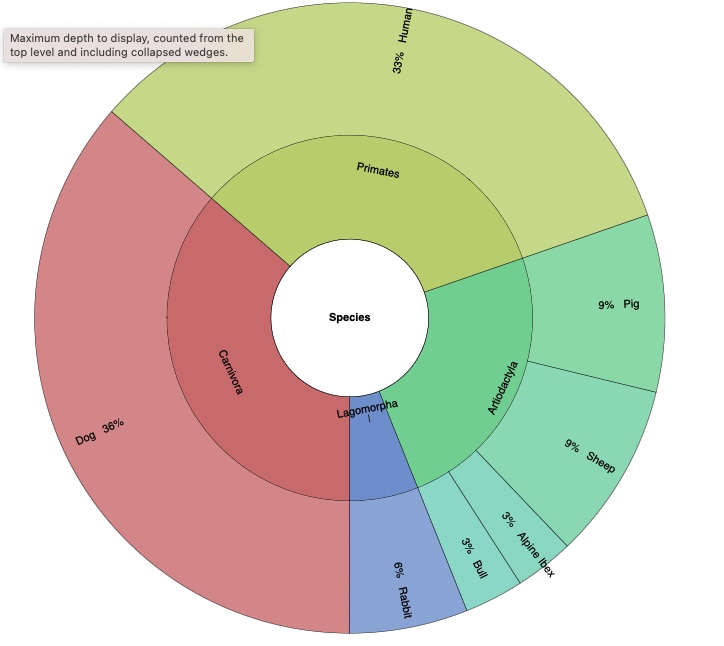


Figure 4. Krona chart of study distribution in type IV hypersensitivity response


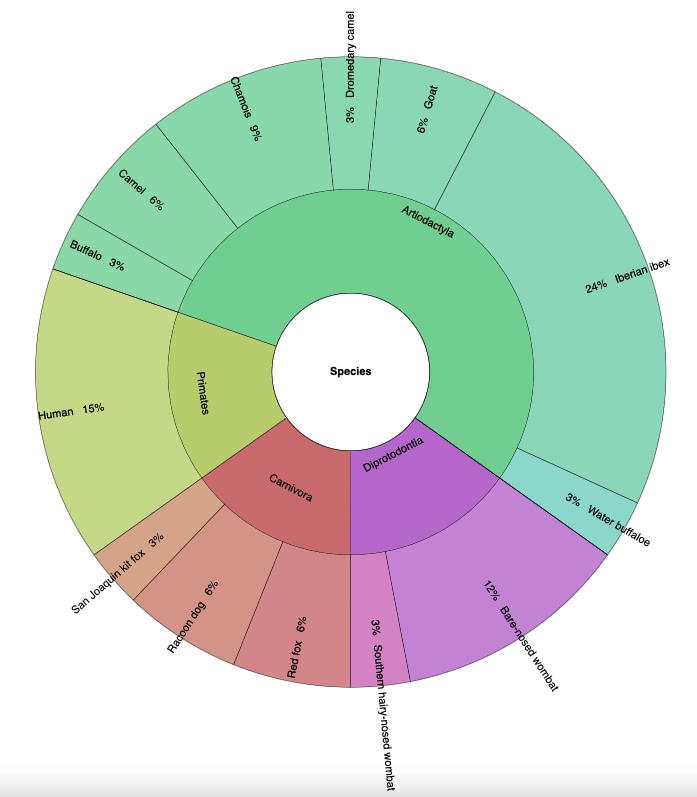

Supplement: Supplementary file 4 — Additional file 4. Figure 1 Flowdiagram & Table 1 Severity groupings. [file 12879_2022_7635_MOESM4_ESM.docx]
