# Supplementary material for: Evidence underscoring immunological and clinical pathological changes associated with Sarcoptes scabiei infection: synthesis and meta-analysis
Source: BMC Infect Dis. 2022 Jul 28;22:658. doi: 10.1186/s12879-022-07635-5 (PMC9335973; doi:10.1186/s12879-022-07635-5)
Supplement: Supplementary file 5 — Additional file 5. Funnel plots and Egger’s regression results [file 12879_2022_7635_MOESM5_ESM.docx]

**Funnel Plots**


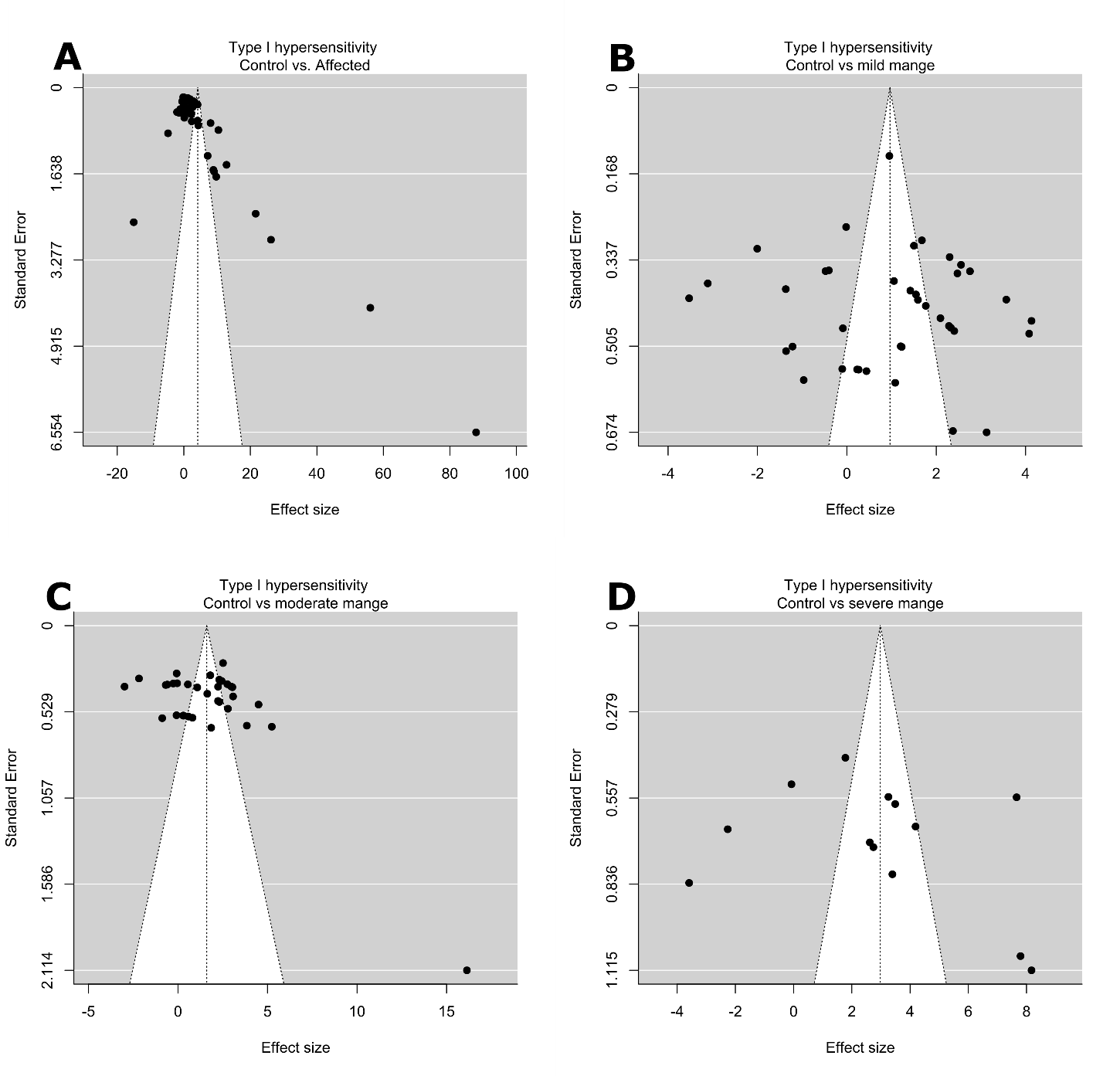


Figure 1. Funnel plots showing the distribution of all effect sizes from host’s experiencing a Type I hypersensitivity response against their respective standard errors for (A) control vs affected; (B) control vs mild mange; (C) control vs moderate mange; and (D) control vs severe mange. Funnel plots are a graphical representation of the dispersion of effect sizes around 0. The vertical black line indicates the mean effect sizes of all points for each plot and the dashed lines indicate the pseudo 95% confidence limits for the heterogeneity. Publication bias is possible due to larger variation in studies with disease severity ranges (B, C, and D).


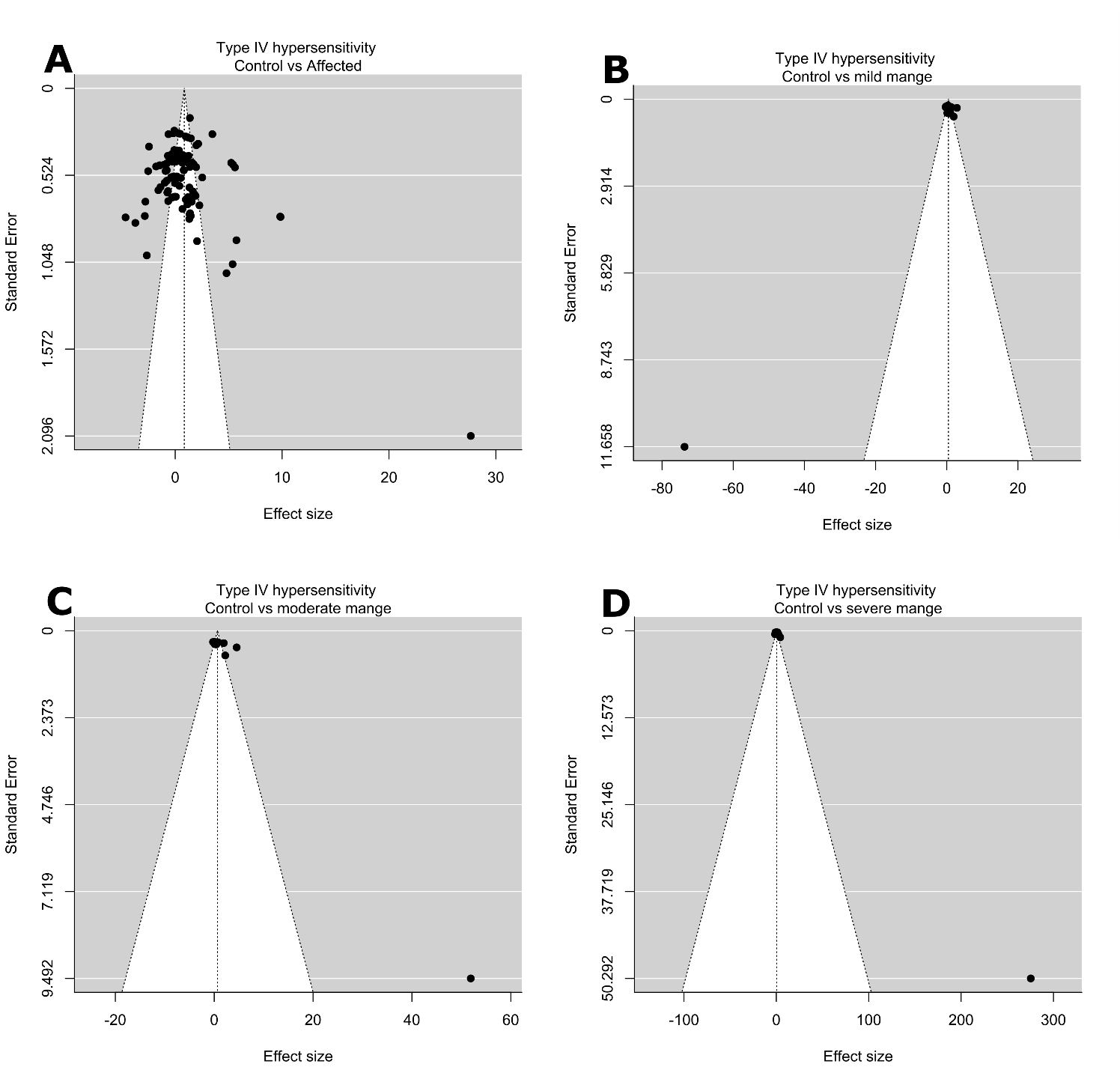


Figure 2. Funnel plots showing the distribution of all effect sizes from host’s experiencing a Type IV hypersensitivity response against their respective standard errors for (A) control vs affected; (B) control vs mild mange; (C) control vs moderate mange; and (D) control vs severe mange. Funnel plots are a graphical representation of the dispersion of effect sizes around 0. The vertical black line indicates the mean effect sizes of all points for each plot and the dashed lines indicate the pseudo 95% confidence limits for the heterogeneity. Publication bias is possible as there is a larger variation in studies with focusing on control vs affected. B, C, and D illustrates that there are fewer studies examining immunological parameters in Type IV hypersensitivity responses.


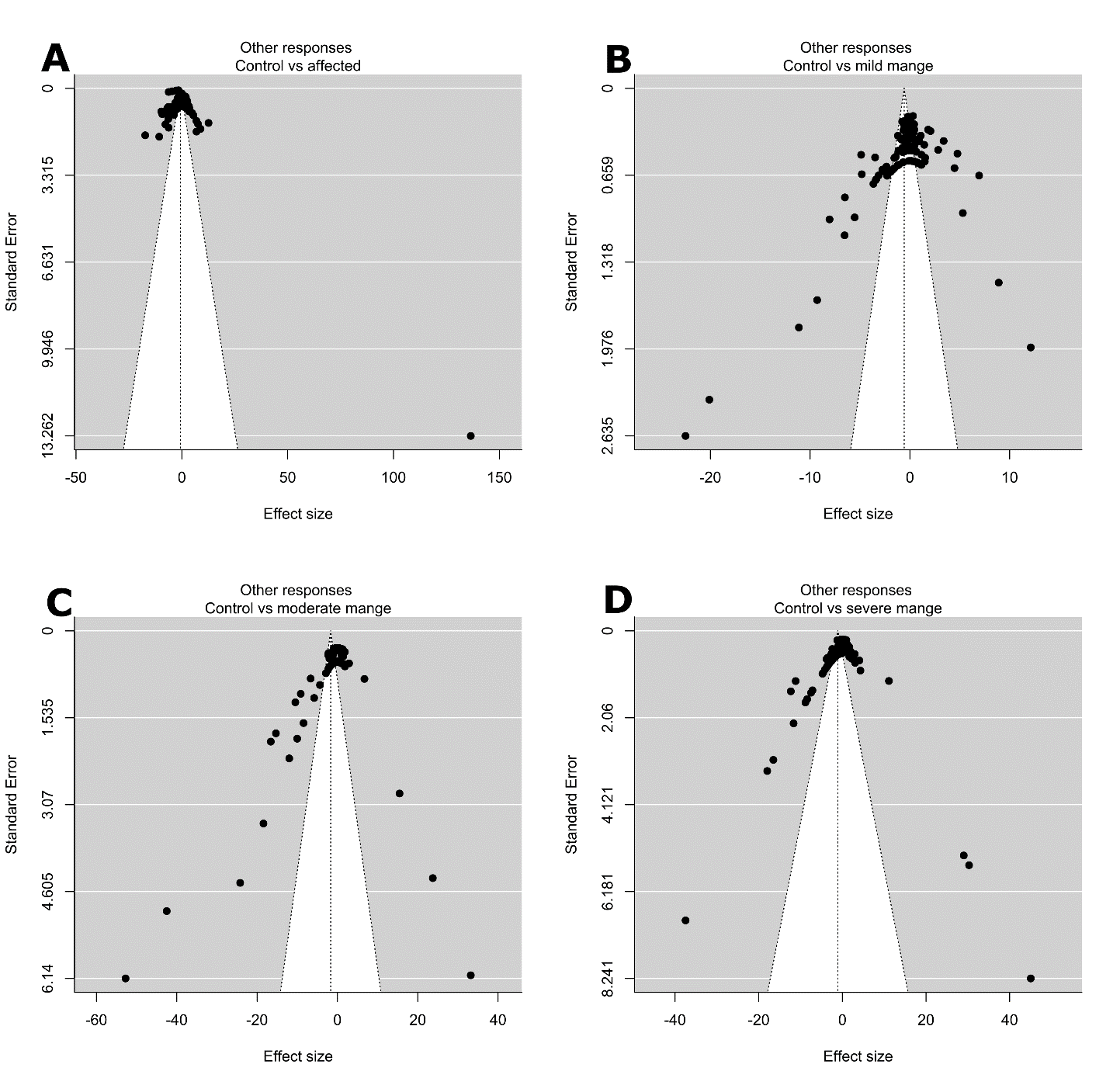


Figure 3. Funnel plots showing the distribution of all effect sizes from host’s experiencing a Type I hypersensitivity response against their respective standard errors for (A) control vs affected; (B) control vs mild mange; (C) control vs moderate mange; and (D) control vs severe mange. Funnel plots are a graphical representation of the dispersion of effect sizes around 0. The vertical black line indicates the mean effect sizes of all points for each plot and the dashed lines indicate the pseudo 95% confidence limits for the heterogeneity. Publication bias is possible as the majority of the values are asymmetrically distributed in the direction of negative results for control vs. mild, moderate and severe mange (i.e. reductions in oxidant/antioxidant status, acute phase protein response, erythrocytic, and hepatological and nephrological changes).

Table 1. Egger's regression for funnel plot asymmetry results for each category (ctrl vs affected, and mild, moderate and severe mange) for Type I hypersensitivity response, Type IV hypersensitivity response and common responses (e.g., oxidant/antioxidant status, acute phase protein response, erythrocytic, hepatological and nephrological changes).

|  | **Ctrl vs Affected** | **Mild mange** | **Moderate mange** | **Severe mange** |
| --- | --- | --- | --- | --- |
| **Type I hypersensitivity** | z = 12.60, p < .0001 | z = 0.48, p = 0.62 | z = 5.17, p < .0001 | z = 1.30, p = 0.19 |
| **Type IV hypersensitivity** | z = 5.79, p < .0001 | z = -4.67, p = < .0001 | z = 6.11, p < .0001 | z = 5.14, p < .0001 |
| **Common responses** | z = 4.03, p < .0001 | z = -6.70, p < .0001 | z = -5.21, p < .0001 | z = 0.62, p = 0.53 |
